# Interventions to Prevent Sexual Harassment Against Nurses—StopSH: Protocol for an Intervention Development Study

**DOI:** 10.2196/71425

**Published:** 2025-10-08

**Authors:** Milena Marta Bruschini, Maria Schubert

**Affiliations:** 1 Institute of Nursing School of Health Sciences ZHAW Zurich University of Applied Sciences Winterthur Switzerland; 2 Faculty of Medicine University of Zurich Zurich Switzerland

**Keywords:** acute care, hospitals, intervention development, nurses, sexual harassment, prevention

## Abstract

**Background:**

Patients’ sexual harassment against nurses is a worldwide phenomenon. Some forms occur on a daily to weekly basis. Despite the known high prevalence and its negative consequences, there is still a lack of evidence-based measures to prevent patients’ sexual harassment against nurses. Given the complexity of the problem, multidimensional interventions are required.

**Objective:**

The main objective of the StopSH project is to develop an evidence-based, complex intervention package to prevent patients’ sexual harassment against nurses and minimize its negative consequences for the acute care sector in the German-speaking part of Switzerland.

**Methods:**

This project is an intervention development study with a multimethod design. It involves the participative development and testing of a complex intervention package in one to two Swiss hospitals as practice partners. The project is carried out in four project phases. First, a systematic scoping review will be conducted to identify and map existing interventions aimed at preventing sexual harassment of nurses or minimizing its consequences. The review will include interventions at the individual, organizational, and network levels of nurses. The problem and needs analysis form the second phase, where a cross-sectional web-based survey will be carried out among nurses in one to two partner hospitals. The aim is to assess the prevalence, forms, and perceived consequences of sexual harassment, as well as existing and desired strategies or support structures. The results will inform the development of the intervention package. As a third phase, a complex intervention package will be codeveloped using a participatory action research approach, based on the findings from the first two phases. This process will involve nurses, hospital management, human resources, and other relevant stakeholders to ensure contextual relevance and feasibility. Finally, during a feasibility assessment, the developed intervention package will be implemented and tested on two to three test wards within the partner hospitals. The mixed methods feasibility study will assess the acceptability, practicality, and preliminary effects of the intervention. Survey data, as well as contextual and observational data, will be collected.

**Results:**

The project was launched in February 2024 and is scheduled to last for 5 years. As of August 2025, this project is in phase 2. Data collection is ongoing. The StopSH project is expected to develop and test a complex intervention package for the prevention of patients’ sexual harassment against nurses. This intervention package is predicted to reduce the prevalence and negative effects of sexual harassment against nurses.

**Conclusions:**

The results of this project will provide important guidance for nurses, but also for their employers, and as such can contribute to the long-term reduction of sexual harassment against nurses. It lays the foundation for the development and adaptation of interventions in further nursing settings and other health care professions.

**International Registered Report Identifier (IRRID):**

DERR1-10.2196/71425

## Introduction

### Background

Sexual harassment toward nurses constitutes a significant issue both internationally and in Switzerland [1-3]. Sexual harassment is defined as any behavior of a sexual or gender-specific nature that is unwelcome on one side and that violates a person’s dignity [4]. It can take place through words (verbal), gestures (nonverbal), or acts (physical) [4,5]. While workplace sexual harassment can occur in many different fields [6,7], health care professionals often report significantly higher rates [8-10], with nurses being among the most frequently affected [11-14].

One reason for nurses being most frequently affected by workplace sexual harassment is [11-14] that patients are among the main perpetrators of sexual harassment in health care [9,11,15,16], and nurses are the professionals who spend the most time directly at the patient’s bedside, performing tasks with close physical contact [17-20]. Further, sexual harassment is often facilitated by power imbalances, and nurses typically work within hierarchical health care systems where they may hold less institutional power compared to other professional groups [14,15,21].

In health care, sexual harassment against nurses can originate from various groups, including patients, patients’ family members, nurse-colleagues, supervisors, and physicians. The primary perpetrators vary across studies, depending on the setting and the cultural context [3,16,22-24]. In studies from the Global North, such as Switzerland, patients are most often identified as the main perpetrators [16,17,23,25]. In contrast, studies from the Global South often report higher rates of harassment by physicians, nurse-colleagues, or patients’ family members [22,24,26-28].

The prevalence of sexual harassment against nurses varies greatly across countries (5%-70%) [23,24,26,27,29,30]. This large range is mainly due to the different measurement methods used in the studies, but also to differences depending on culture, country, and setting [2,3]. Furthermore, the issue of sexual harassment is still often associated with shame, which can lead to incidents not being reported and therefore being undetected [3,17,22,31,32]. A recent study conducted in the German-speaking part of Switzerland, using a newly developed sensitive instrument by Vincent-Höper et al [5] and adapted by Adler et al [29], revealed an alarming prevalence: 95.6% of nurses reported experiencing at least one form of sexual harassment from patients in the past 12 months. Some forms were encountered on a weekly or even daily basis [1].

Sexual harassment against nurses has various negative effects on those affected, but also on the institution and the quality of care provided to patients. Almost 70% of nurses affected report negative psychological and physical consequences [26,33], such as anxiety, humiliation, frustration, gastrointestinal complaints, headaches, insomnia, and exhaustion. In addition, several studies have confirmed negative consequences on the work performance, work motivation, and quality of care of nursing staff [15,17,22,23,26,34].

Poor working conditions, emotional stress, and workplace violence have been shown to affect nurses’ retention in the profession [35,36]. As seen globally, the shortage of nursing staff is also a prevailing issue in Switzerland, which can be exacerbated by workplace sexual harassment [37,38]. Studies have shown that sexual harassment can increase the pressure on nurses to such an extent that they go on sick leave, resign, or even change jobs. This is particularly the case if this occurs on a recurring basis [17,22,25,26,39].

The prevalence of sexual harassment against nurses, the groups of perpetrators, influencing factors, and forms, as well as the behavioral patterns of nurses and the negative consequences, have been extensively researched worldwide [2,3,15,34]. However, there are currently no universally applicable recommendations for strategies regarding this issue [40], and the development of evidence-based, tailored interventions and guidelines for health care facilities is deemed necessary [15,41-43]. Given the complexity of the problem, it is clear that there is no one-size-fits-all solution and that multidimensional interventions are required, including the individual, organizational, and network level [40,44-46].

Research results on specific measures to prevent patients’ sexual harassment against nurses and minimize its negative effects are lacking. The StopSH project aims to fill this research gap. The project will focus on the acute care sector in the German-speaking part of Switzerland and the perpetrator group of patients. The focus on patients and acute care is partly due to the fact that in countries of the Global North, including Switzerland, patients are the main perpetrator group [9,16,23,25], and about half of the nursing staff in Switzerland work in hospitals [47]. Furthermore, studies report that acute care nurses experience sexual harassment more frequently than nurses in other sectors [1,3].

### Objectives

The main objective of the StopSH project is to codevelop an evidence-based, complex intervention package to prevent patients’ sexual harassment against nurses and minimize its negative consequences for the acute care sector in the German-speaking part of Switzerland. The aim is to enable health care organizations, nurses, and their network to take action against sexual harassment.

The main research question of this project is, which individual, organizational, and network interventions to prevent patients’ sexual harassment of nurses and minimize its negative consequences are considered feasible and applicable in Swiss hospitals by key stakeholders involved in the participatory development process?

In addition to the overarching question, three subquestions were formulated to achieve the objective: (1) What evidence-based interventions to prevent workplace sexual harassment against nurses and minimize its negative consequences at the individual, organizational, and network levels of nurses are described in the literature? (2) What problems and needs do stakeholders from Swiss hospitals state regarding the prevention of patients’ sexual harassment of nurses and the minimization of its negative consequences? (3) Which interventions at individual, organizational, and network levels are suitable, feasible, and acceptable for the prevention of patients’ sexual harassment of nurses and the minimization of its negative consequences in Swiss hospitals, based on stakeholder input and feasibility testing?

Each subquestion is addressed in a dedicated study phase and outlined in the Methods section.

## Methods

### Project Design and Phases

The StopSH project is an intervention development study with a multimethod design. It involves the development and testing of a complex intervention package to prevent patients’ sexual harassment of nurses in hospitals located in the German-speaking part of Switzerland. This intervention development process will be carried out participatively with one to two Swiss hospitals in the German-speaking part of Switzerland as practice partners. The hospitals will be selected based on availability and willingness to participate. Inclusion criteria comprise a high level of motivation to address the issue of sexual harassment, a commitment to long-term collaboration with the research team, and an interest in sustainably implementing the intervention package.

Conducting the project in collaboration with one to two practice partners allows for a focused and resource-efficient development process, enabling close collaboration with stakeholders and iterative refinement of the intervention package. In follow-up projects, the intervention package can be adapted and implemented in additional hospitals and care settings to increase its reach and applicability.

**Figure 1:**
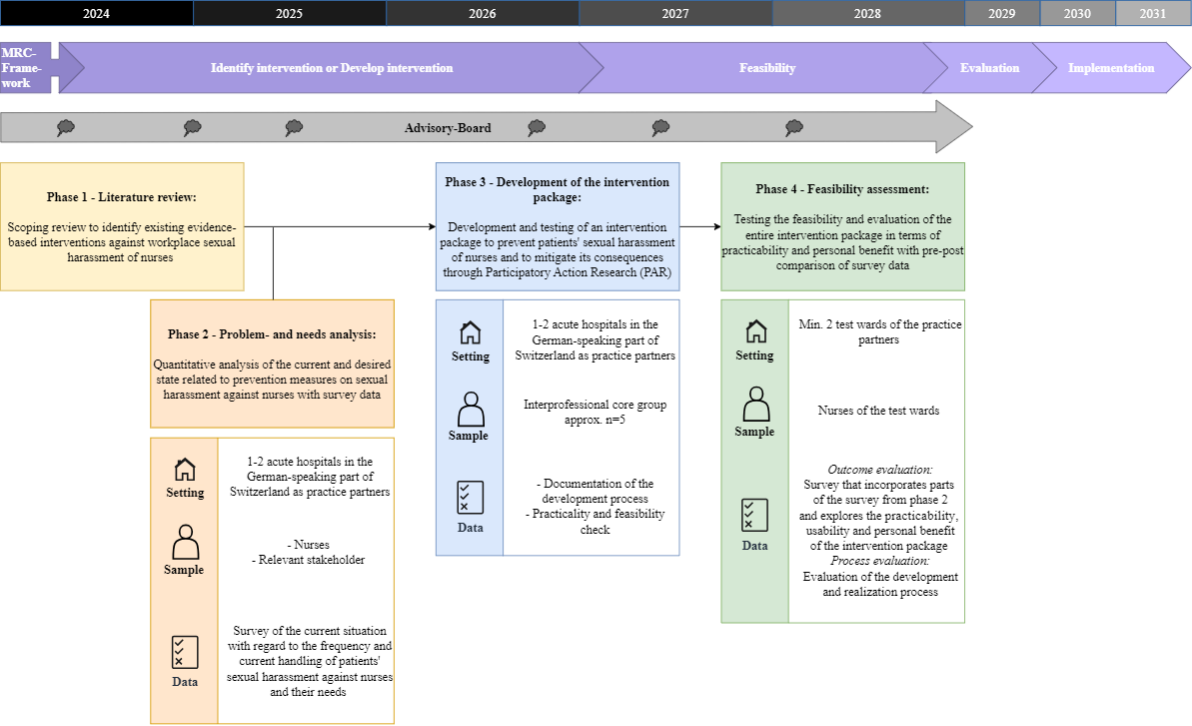
Overview of the StopSH project phases with reference to the MRC framework. MRC: Medical Research Council.

The development and testing of a complex intervention package to prevent and minimize the negative consequences of patients’ sexual harassment against nurses is carried out in four project phases (Figure 1):

- Phase 1: literature review
- Phase 2: problem and needs analysis
- Phase 3: development of the intervention package
- Phase 4: feasibility assessment

To support the participatory development process, an interprofessional core group of approximately five key stakeholders of the practice partners will be formed. This core group may include nursing staff, vocational trainers, people from the field of quality development and psychology, nursing managers, and human resources representatives. Furthermore, the inclusion of an advisory board ensures the participation of key stakeholders from the Swiss health care system and field experts throughout the development process. This structure ensures that the intervention package is both contextually relevant and feasible for the practice partners and facilitates later broader implementation in the Swiss acute care sector. The interprofessional core group will be actively involved in phases 3 and 4 of the project, contributing to the participatory development and feasibility testing of the intervention package. The advisory board advises the project throughout all four phases and meets twice a year. It provides strategic input, supports scientific and contextual alignment, and ensures that the intervention package remains relevant beyond the immediate hospital setting.

The Medical Research Council (MRC) framework [48-51] forms the methodological framework for project implementation and the overall structure of the project. StopSH covers the MRC phases “identify or develop intervention” and “feasibility,” while the phases “evaluation” and “implementation” are beyond its scope and will be addressed in follow-up projects. The “Conceptual Framework for Systematic Capacity Strengthening for Health Policy and Systems Research (HPSR)” [46] forms the conceptual framework for realization. In this project, the Conceptual Framework for Systematic Capacity Strengthening for HPSR [46] provides the conceptual foundation for the participatory research approach and the development of appropriate interventions to prevent patients’ sexual harassment of nurses in Swiss hospitals. It emphasizes the importance of working with shared goals, values, and principles, while addressing all three capacity levels: individual, organizational, and network. The key components of the Conceptual Framework for Systematic Capacity Strengthening for HPSR [46] are reflected in the objectives and design of each phase. Both frameworks are suitable for the development of complex interventions and emphasize the importance of addressing all relevant levels (individual, organizational, and network) to ensure effectiveness and sustainability. Complexity is addressed through several strategies: first, the intervention is designed as a multicomponent package, incorporating measures at the individual level (eg, training for nurses), organizational level (eg, reporting structures), and network level (eg, collaboration with external stakeholders). Second, the participatory approach ensures that the intervention package is tailored to the specific needs and contextual conditions of the practice partners, allowing for iterative refinement based on stakeholder feedback.

### Phase 1: Literature Review

In the context of the Conceptual Framework for Systematic Capacity Strengthening for HPSR [46], the evidence-based literature is an important basis for informed decision-making [46,52]. Accordingly, phase 1 forms the basis of the StopSH project, providing the evidence base that will inform both the survey in phase 2 and the codevelopment of the intervention package in phase 3. This phase aims to identify existing, evidence-based intervention measures against workplace sexual harassment of nurses, without restrictions regarding the type of perpetrator or care setting. Using a scoping review design, the state of the literature will be determined (subquestion 1).

All types of evidence published in English or German will be included, provided they focus on interventions addressing workplace sexual harassment of nurses in any setting. No restrictions will be applied regarding the publication date. The literature search will be conducted in medical databases such as MEDLINE, CINAHL, and PsycInfo. In addition, supplementary searches will be performed using Google Scholar and trial and guideline registries, as well as forward and backward citation tracking. Included studies will undergo a thematic analysis and a numerical summary to identify existing interventions and highlight potential research gaps. In line with scoping review methodology [53], no critical appraisal of included studies will be conducted, as the aim is to map the existing evidence rather than evaluate their quality. Further information on the scoping review can be found in the published protocol on the Open Science Framework by Bruschini et al [54]. The reporting of the scoping review will follow the PRISMA-ScR (Preferred Reporting Items for Systematic Reviews and Meta-Analyses extension for Scoping Reviews) guideline as outlined by Tricco et al [55].

### Phase 2: Problem and Needs Analysis

#### Overview

According to the Conceptual Framework for Systematic Capacity Strengthening for HPSR [46], the process of improvement starts with an assessment of the current situation and needs. It is important to involve different people in this process in order to obtain a collective picture of the situation and current capacities. Therefore, project phase 2 aims to carry out a problem and needs analysis with nurses and other relevant stakeholders of the practice partners (subquestion 2).

#### Design and Setting

The problem and needs analysis will be carried out using a cross-sectional design. A quantitative web-based survey will be conducted in one to two hospitals as practice partners in the German-speaking part of Switzerland. The results from phase 1 will contribute to the development of the survey, with a particular focus on identifying existing interventions and those that nurses would like to see in the future.

#### Participants and Sampling

Nurses of the participating practice partners, who are working in direct patient care, will be invited to participate in the survey. There will be no restrictions regarding nurses’ age, gender, years of professional experience, or level of education. For each participating hospital, the aim is to conduct a full census of all eligible nurses. Depending on the size and structure of the participating hospitals, the inclusion of all departments may not be feasible. In such cases, a convenience sampling strategy will be applied in collaboration with the practice partners to ensure the inclusion of relevant departments.

In addition to nurses, other relevant stakeholders, such as nurse managers, vocational trainers, psychologists, and human resources personnel employed by the practice partners who have prior experience in dealing with sexual harassment of nurses and are involved in safeguarding employee integrity or managing crisis situations, will also be invited to participate in the survey. As the study aims to conduct descriptive analyses only and follows a full census approach, no a priori sample size calculation is performed. The research team aims to recruit a minimum of 100 nurses and 10 other relevant stakeholders for the survey.

#### Data Collection and Analysis

##### Overview

Data collection will be conducted via REDCap (Research Electronic Data Capture; Vanderbilt University) [56,57] in a cross-sectional manner over a period of 1 to 2 months per practice partner. Recruitment and data collection will follow a stepwise approach. Participants will receive a survey link via the internal email system of the practice partners. The survey will consist of approximately 80 items and include the following components.

##### Section A (Current State)

Items assessing the prevalence, impact, and influencing factors of sexual harassment. This includes the full, reliable and validated (Cronbach α 0.80 to 0.92) “Sexually Harassing Behavior Questionnaire from an Extraorganizational Perspective” (SHBQ-X) [5,29], as well as selected items from a Swiss national survey on workplace sexual harassment developed by the Federal Office for Gender Equality (FOGE) and the State Secretariat for Economic Affairs (SECO) [10].

##### Section B (Desired State)

This section includes adapted items from the FOGE/SECO survey and newly developed items based on findings from phase 1. It focuses on intervention needs, desired preventive interventions, and perceived barriers and facilitators for implementation.

##### Section C (Demographics)

These items will collect information about the participants’ age, gender, education, and professional experience. They will be used for the description of the participants.

All instruments used in the survey are available in German, which is the language of the study. Permissions to use the SHBQ-X and the FOGE/SECO items were obtained from the original authors [5,10,29].

To ensure content validity, the created survey for phase 2 will undergo a content validation process following Yusoff [58], involving 6-10 experts (including advisory board members, representatives, and nurses of the practice partners). Content Validity Index values will be calculated for each item [58]. The data will be analyzed descriptively using the programming language and environment R (R Core Team) and RStudio (Posit Software, PBC).

### Phase 3: Development of the Intervention Package

#### Overview

The “Strengthening and unleashing” process of the Conceptual Framework for Systematic Capacity Strengthening for HPSR [46] aims to strengthen capacities at all three levels (individual, organizational, and network), taking into account identified resources and needs, as well as values and principles. The third phase will develop an intervention package to prevent sexual harassment against nurses and minimize its negative consequences. This will be accomplished in a participatory manner together with nurses and other relevant stakeholders in the selected one to two hospitals (interprofessional core group). In this phase, single interventions for the intervention package will be developed, tested, and adapted for everyday use (subquestion 3).

#### Design and Setting

Phase 3 is based on the participatory action research approach. Specifically, the plan-do-study-act (PDSA) process by Deming [59] and adapted by Magnuson et al [60] will be used to develop, test, and adapt the prototype of the intervention package. The intervention package will be developed and tested in a participatory manner with the practice partners and the research team. Further, the process will be supported by the advisory board. The results from phases 1 and 2 will inform the development process by identifying interventions that are both evidence-informed, contextually appropriate, and preferred by nurses in the partner hospitals. The previously gained knowledge will serve as the foundation for codeveloping an intervention package tailored to the specific needs and realities of the practice partners.

#### Participants and Sampling

The practice partners’ interprofessional core group will participate in the PDSA process and collect data while developing and testing the single interventions. Depending on the specific focus of each intervention within the package, key stakeholders from the practice partners and advisory board will be identified, and the core group will be assembled accordingly. The composition of the core group will remain flexible to ensure that the expertise required for each single intervention is adequately represented.

#### Data Collection and Analysis

Each intervention undergoes the PDSA process individually. The process is repeated until a customized intervention package is available. During the development process, documentation and observation data are collected. This is done using documentation forms, meeting and discussion minutes, implementation logs, and reflection diaries. This data is used to document the development process, support decision-making, and contribute to the process evaluation in the subsequent project phase 4.

To determine the success of each PDSA cycle, predefined criteria will be established for each intervention component. These will include formative process indicators (eg, feasibility, acceptability, and initial fidelity) and outcome-oriented perceptions (eg, perceived relevance, usability, and practicability). Progression to the next cycle or finalization of an intervention will be based on whether these criteria are met, as assessed through documentation and feedback from the core group and advisory board.

The final intervention package is expected to comprise approximately three to five single interventions. This range is based on practical considerations, including available resources and the intention to ensure a manageable scope of change within the clinical setting.

### Phase 4: Feasibility Assessment

#### Overview

Although the Conceptual Framework for Systematic Capacity Strengthening for HPSR [46] does not explicitly refer to the step of intervention testing and evaluation, this phase can be understood as part of the “unleashing” process. The MRC framework [48-51] emphasizes the importance of testing interventions within the given context and evaluating both the process and the outcome. Therefore, in this phase, the feasibility, benefit, and acceptance of the entire intervention package developed will be tested and evaluated. Project phase 4, together with phase 3, serves to answer subquestion 3.

#### Design and Setting

This feasibility study serves to evaluate the entire intervention package (outcome and process evaluation). The outcome evaluation does not yet test the effectiveness of the intervention package regarding the prevention of patients’ sexual harassment and minimization of its negative consequences, but serves to evaluate the feasibility, practicability, and personal benefit of the developed intervention package. The application of the intervention package prototype and the feasibility assessment are carried out on at least two test wards of the practice partners.

#### Participants and Sampling

For the evaluation process, all nurses with direct patient contact working on the test wards are invited to participate in a second survey. Some of the participants may be the same as in the first survey conducted in phase 2. This overlap is likely, as phase 2 may involve a full census of all nursing staff employed at that time. While prior participation can lead to increased sensitization regarding the topic of sexual harassment, this cannot be fully controlled due to the anonymous nature of the survey and the possibility of informal exchange among nurses. Consequently, even those who did not participate in phase 2 may have gained knowledge about the topic. Since phase 4 focuses on assessing feasibility, usability, and perceived benefit rather than effectiveness, differences in prior exposure to the survey or study topic are not considered problematic.

#### Data Collection and Analysis

To explore early indications of the intervention package’s performance and to describe possible changes (outcome evaluation), a before-and-after comparison will be carried out using a quantitative web-based survey. The second survey includes items from Section A of the phase 2 survey, aiming to reassess the prevalence, impact, and influencing factors of sexual harassment. Section A comprises the validated SHBQ-X questionnaire [5,29] and selected items from the Swiss national survey on workplace sexual harassment [10].

The quantitative baseline data from project phase 2 will be compared with data collected 3 to 6 months after the application of the intervention package on the test wards. To account for potential differences in prior exposure to the study topic, participants will be asked whether they have already taken part in the initial survey conducted in phase 2. This information will be used to explore possible variations in responses between returning and new participants.

The before-and-after comparison is exploratory in nature and aims to identify early indications of change. Descriptive subgroup analyses will be conducted to explore potential variations in responses between returning and new participants. In addition, the second survey includes items specifically designed to assess the intervention package’s perceived benefits for nurses, as well as its practicability and usability.

For the process evaluation, contextual and observational data on the implementation setting will be collected by the research team. This documentation and observation data from phases 3 and 4 will be analyzed to assess the fidelity of implementation in relation to the planned intervention package.

Quantitative data will be analyzed descriptively using the programming language and environment R and RStudio (Posit Software, PBC). Textual and documentation data for process evaluation are analyzed using MAXQDA (VERBI Software) based on inductive content analysis according to Kyngäs [61] and Elo and Kyngäs [62].

### Ethical Considerations

The planned project was submitted to the Cantonal Ethics Committee of Zurich (BASEC—Nr. Req-2024-01085). In accordance with Article 2, the research project does not fall within the scope of the Swiss Human Research Act [63], as it does not investigate questions related to human diseases or the structure and function of the human body, nor does it involve the analysis of health-related data or biological material. The research project complies with Swiss law and the ethical principles of the Declaration of Helsinki [64]. Participation in the study is voluntary and does not involve any risk for the participants. No financial compensation is provided for participation. Nurses who do not take part in the study do not experience any disadvantage. All study participants must give their consent to participate in the study. In the web-based survey, consent is indicated by placing a cross in the checkbox provided. For core group participants, written informed consent will be obtained using a consent form prior to their participation. Due to the sensitive nature of the topic, participants are provided with contact details for counseling centers regarding workplace sexual harassment and have the option of withdrawing their consent to participate in the study at any time. The study is designed to minimize emotional burden by avoiding open-ended questions or detailed descriptions of personal experiences. Participants are asked to respond to predefined categories (eg, types of sexual harassment encountered and perceived consequences). The main focus lies on the development of prevention measures. Further, during data collection, particular attention is paid to the voluntariness of responses. With the exception of the consent form and eligibility criteria at the beginning of the survey, none of the questions are mandatory, and participants may skip any items they do not wish to answer. Data collection in phases 2 and 4 is done anonymously via the electronic web app REDCap [56,57].

### Confidentiality and Availability of Data and Materials

The protection of sensitive data is guaranteed throughout the entire research process. Personal information is stored separately from the research results. All data are stored in a secure, password-protected folder at the study site, with the server located in Switzerland and archived for 10 years. All study and participant data will be treated with the utmost discretion and will only be accessible to members of the research team. In the planned scientific publications, the data will be presented in such a way that no conclusions can be drawn about individuals or single institutions. Data will either be collected anonymously or be pseudonymized during data collection, by assigning participant codes (with the key stored separately) and removing directly identifying information.

The collected data will not be passed on to third parties. However, metadata that support the findings of this project will be made publicly available via a public repository.

### Dissemination Policy

The StopSH project aims to produce three scientific publications. For this purpose, the authorship eligibility guidelines of each journal will be followed. The results will be published with open access. Furthermore, study results will be communicated at public lectures and congresses for health care professionals. Study results are also made available to the public in easy language.

## Results

### Current Status of the Project

This 5-year project was initiated in February 2024. As of August 2025, this project is in phase 2. In this regard, the data collection of phase 2 via web-based survey is ongoing, with approximately 50 participants recruited so far from one practice partner. Furthermore, preparations are underway for the publication of phase 1 (scoping review).

The project was originally planned to run for 3 years. However, in August 2025, due to the current recruitment status and the absence of secured third-party funding, the research team decided to extend the duration to 5 years to ensure sufficient time for completion of all phases.

### Expected Results

The StopSH project will develop and test an evidence-based, complex intervention package for the prevention of patients’ sexual harassment against nurses. This will be achieved through close collaboration with the practice partners and the advisory board. The intervention package can empower health care organizations, nurses, and their network to take action against sexual harassment, thereby improving the workplace safety of nurses in Switzerland. In the long term, the intervention package is expected to reduce the prevalence and negative effects of sexual harassment against nurses. Improved working conditions can enhance work satisfaction and reduce the number of nurses leaving the workforce early.

## Discussion

### Principal Findings

This study protocol refers to a project that aims to make a significant contribution to the prevention of patients’ sexual harassment of nurses. The StopSH project provides an overview of potential preventive interventions and offers insights into the current and desired future state regarding prevalence and prevention of workplace sexual harassment in the acute care setting of the German-speaking part of Switzerland. It also transparently documents the development process of evidence-based interventions and evaluates their feasibility. As such, the StopSH project lays an important foundation for addressing patients’ sexual harassment in health care settings and can serve as a model for similar initiatives in other health care sectors.

The #MeToo and #EndNurseAbuse movements have drawn national and international attention to the issue of workplace sexual harassment in nursing [32,65-67]. Increased media coverage in recent years has raised public and political awareness of this issue in Switzerland. This heightened awareness forms the basis for the realization of this project. Despite this increased awareness, the sensitive nature of the topic may still pose challenges during implementation. Discussing experiences of sexual harassment can evoke discomfort or fear of stigmatization among participants [15,25,29]. The research team is therefore prepared to address potential resistance or hesitancy by fostering a safe and respectful environment, ensuring anonymity, and providing access to support services. These considerations are essential to promote trust and engagement, and to ensure the feasibility of the intervention package in real-world health care settings.

Given the high prevalence of sexual harassment against nurses [23,24,26,27,29,30] and the lack of effective prevention [15,41-43], there is an urgent need for targeted interventions. Ensuring the safety of nursing staff is essential for the sustainable development of the profession. Research has shown that improving working conditions and protecting the health of nurses positively influences retention in the profession [68]. By codeveloping an intervention package, this project addresses a critical research gap and responds to an urgent need identified in clinical practice.

StopSH contributes to improving working conditions in nursing and further promotes awareness of the issue. The involvement of the practice partners, experts, and nurses from the outset ensures that interventions are aligned with practical needs and are feasible. According to Cook et al [20], a participatory approach is essential for developing effective interventions against patients’ sexual harassment of nurses, as it enables an understanding of the complexity, dilemmas, and relationship values held by nurses.

### Strengths

This project, which aims to develop evidence-based prevention measures for sexual harassment in the nursing profession, is the first of its kind in Switzerland. It can contribute to achieving the goals of the Swiss nursing initiative, a program aimed at nationally improving working conditions, enhancing nursing education, and increasing the attractiveness of nursing professions [69].

The project’s added value is highlighted by the cocreation of a specific intervention package designed to prevent sexual harassment against nurses in the acute care sector and mitigate its negative consequences, considering all three capacity levels as outlined by Mirzoev et al [46].

### Limitations

The implementation of the participatory action research design is dependent on the active participation of the practice partners and stakeholders. This makes the success of the project reliant on the commitment, resources, and motivation of the practice partners. Shortages of health care staff and resources are also ubiquitous in Switzerland, which poses obstacles for the project. For this reason, the development of the intervention package must be adapted to the resources of the respective practice partners. To mitigate these resource-related limitations, the research team is seeking third-party funding to compensate the practice partners. In addition, the research team will be preparing all study materials and communication templates to reduce the administrative burden of the practice partners.

This project focuses on nursing staff working in one to two hospitals in the German-speaking part of Switzerland. This specific focus and small sample do not allow the generalization of the results to other health care settings. Furthermore, as the study is designed for descriptive and developmental purposes only, no causal inferences can be drawn from the findings. Nevertheless, the close collaboration with one to two practice partners enables a context-sensitive and resource-efficient development process. The findings and intervention package will serve as a foundation for future projects aiming to adapt and evaluate the intervention package in broader health care contexts.

The voluntary nature of participation and recruitment in the project may introduce selection bias. Individuals who are particularly affected by workplace sexual harassment might be more inclined to participate due to personal relevance, or alternatively, they might choose not to participate due to emotional burden. Despite the possibility of either underrepresentation or overrepresentation of those affected, voluntary participation is considered ethically appropriate given the sensitive nature of the topic.

Furthermore, demonstrating the long-term effects of the intervention package regarding the prevention of patients’ sexual harassment and minimization of its negative consequences, as well as the implementation process, is beyond the scope of the StopSH project and will be addressed in a follow-up project (longitudinal intervention study).

### Conclusions

Maintaining a respectful, nonviolent work environment is key to keeping nurses in the profession. The results of this project will provide important guidance for nurses, but also for their employers. It may contribute to the long-term reduction of sexual harassment against nurses and therefore sustainably improve workplace safety.

## Abbreviations

FOGE: Federal Office for Gender Equality
HPSR: Health Policy and Systems Research
MRC: Medical Research Council
PDSA: Plan-do-study-act
PRISMA-ScR: Preferred Reporting Items for Systematic Reviews and Meta-Analyses extension for Scoping Reviews
REDCap: Research Electronic Data Capture
SECO: State Secretariat for Economic Affairs
SHBQ-X: Sexually Harassing Behavior Questionnaire from an Extraorganizational Perspective
